# Giant Chorioangioma of the Placenta as a Cause of Maternal, Foetal, and Neonatal Complications

**DOI:** 10.7759/cureus.42430

**Published:** 2023-07-25

**Authors:** Akshata Mestha, Arva Dhanaliwala, Antoine Frangieh, Suvarna M Mestha, Sridhar M Ramaiah

**Affiliations:** 1 General Practice, Dubai Academic Health Corporation, Dubai, ARE; 2 Obstetrics and Gynaecology, American Hospital, Dubai, ARE; 3 Neonatology, American Hospital, Dubai, ARE

**Keywords:** foeto-maternal complications, pregnancy outcome, benign tumor, placental neoplasm, chorioangioma

## Abstract

Chorioangioma is a benign placental neoplasm seen in about one percent of all pregnancies. The larger neoplasms generally cause severe foeto-maternal complications. We are reporting a case of a 33-year-old gravida three para two female who was incidentally diagnosed with chorioangioma at her routine 28-week antenatal follow-up. She delivered a preterm small-for-gestational-age female baby at 34 weeks with complications. Therefore, an early diagnosis warrants a close follow-up and timely intervention for a better outcome of the pregnancy.

## Introduction

Chorioangioma is a benign non-trophoblastic placental neoplasm that develops from chorionic mesenchyme due to atypical development of blood vessels [1-4]. It is usually seen in the last trimester of the pregnancy over the foetal part of the placenta [3]. It occurs in about one percent of all pregnancies [2,4-8]. The smaller tumors (less than 5 cm) are common and generally insignificant whereas the larger ones (more than 5 cm) are rare (1:9000 - 1:50000) [2,6], but they are capable of causing severe complications for the mother and baby despite being diagnosed by antenatal ultrasound (USS) [2-5,7].

## Case presentation

A 33-year-old female, gravida three para two with previous two term pregnancies delivered via normal vaginal delivery without any complications came for her regular 28-week follow up where her USS showed a well-circumscribed lesion with blood flow and low-level internal echoes measuring 9.79 cm x 7.45 cm at the upper end of the placenta. There was a single healthy foetus with an estimated foetal weight (EFW) of 1280 g and an amniotic fluid index (AFI) of 15 cm.

**Figure 1:**
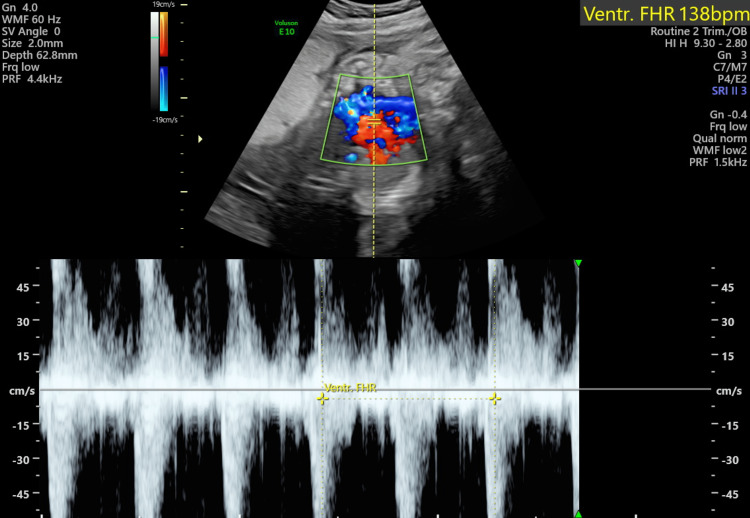
Foetal heart rate (FHR) at 30 weeks gestation

**Figure 2:**
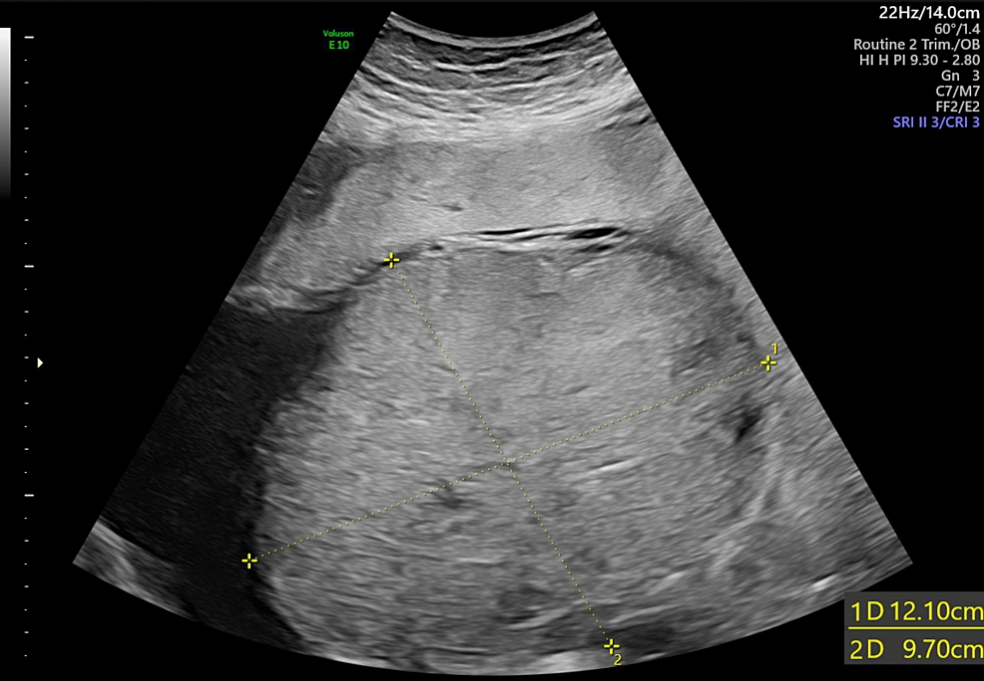
Size of the chorioangioma at 30 weeks pregnancy

**Figure 3:**
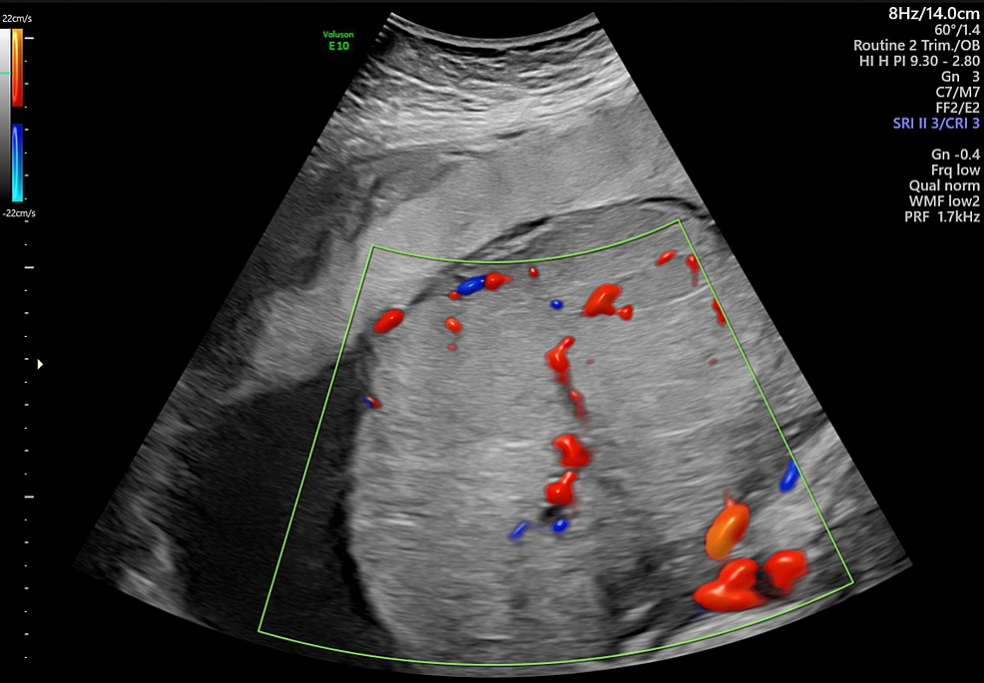
Blood supply of the chorioangioma The tumor is vascularized by the umbilical blood vessels.

**Figure 4:**
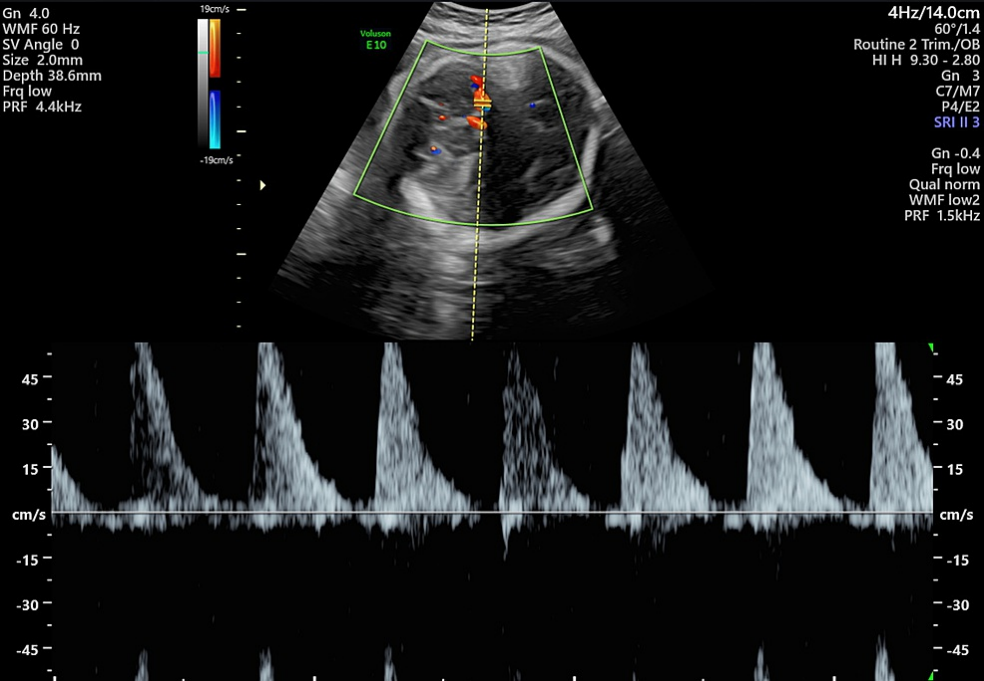
Peak systolic velocity of the middle cerebral artery of the foetus at 30 weeks gestation

She was referred to the foeto-maternal medicine unit (FMMU) for a detailed USS scan which showed a single intrauterine pregnancy with cephalic presentation and an AFI of 11 cm. The measurements were appropriate for 31 weeks and EFW was 1673 g. Fetal heart rhythm was normal and heart rate was 138 bpm (Figure 1). An echogenic mass measuring 12.1 cm x 9.7 cm (Figure 2) was located underneath the chorionic plate near the insertion of the umbilical cord and protruding through the amniotic cavity which was highly vascularized (Figure 3) and was suggestive of a chorioangioma. The middle cerebral artery (MCA) peak systolic velocity (PSV) was 53 cm/sec (Figure 4), which was suggestive of mild anemia. 

**Figure 5:**
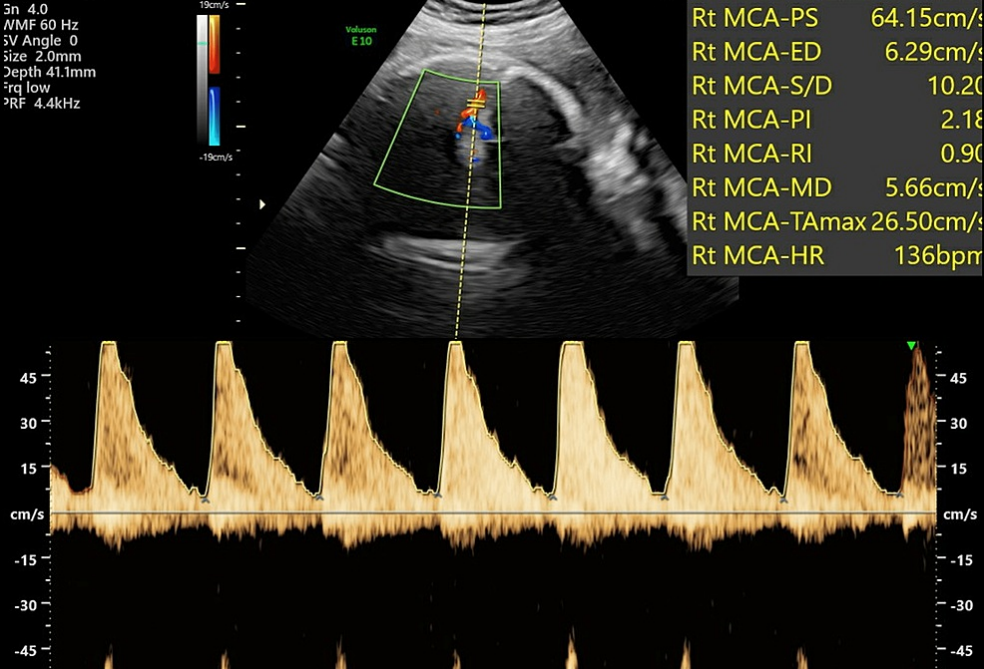
Peak systolic velocity of the middle cerebral artery (MCA) of the foetus at 32 weeks gestation

**Figure 6:**
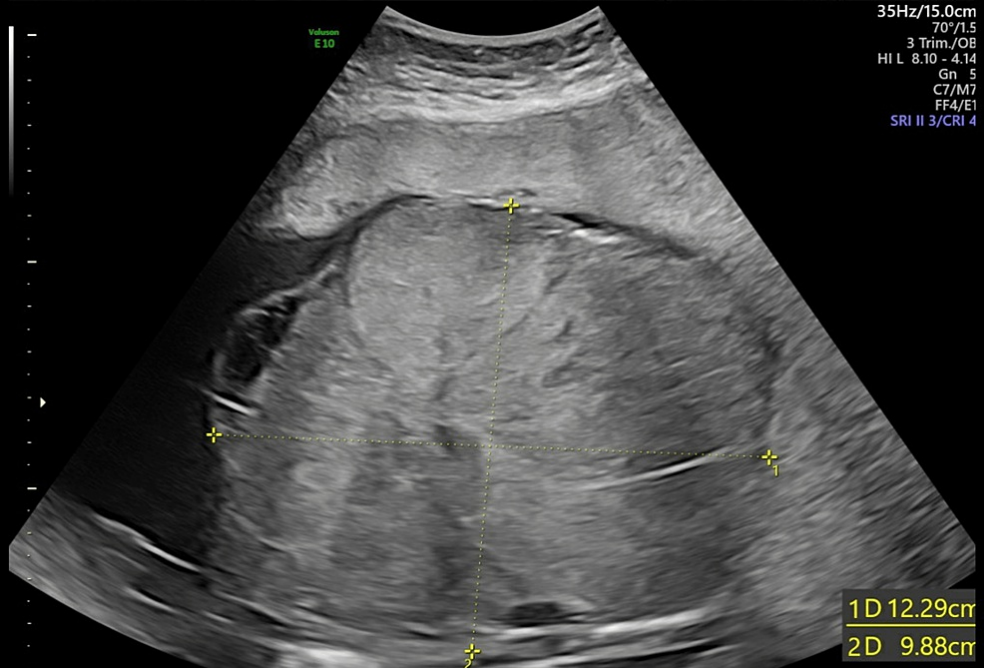
Size of the chorioangioma at 32 weeks pregnancy

Her follow-up USS scan at 32 weeks at the FMMU showed foetal measurements appropriate to gestational age (GA) with EFW of 1892 g and AFI of 11 cm. The PSV of the MCA was 64.15 cm/sec (Figure 5) indicating mild to moderate anemia. The placental neoplasm slightly increased in size and was measuring 12.29 cm x 9.88 cm (Figure 6).

**Figure 7:**
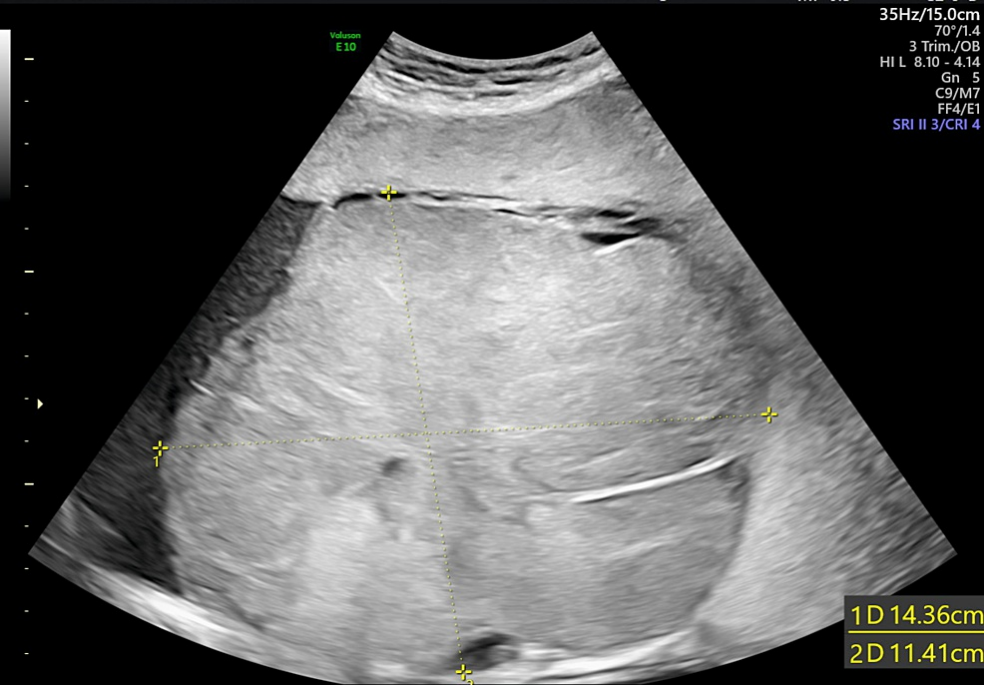
Size of the chorioangioma at 33 weeks pregnancy

**Figure 8:**
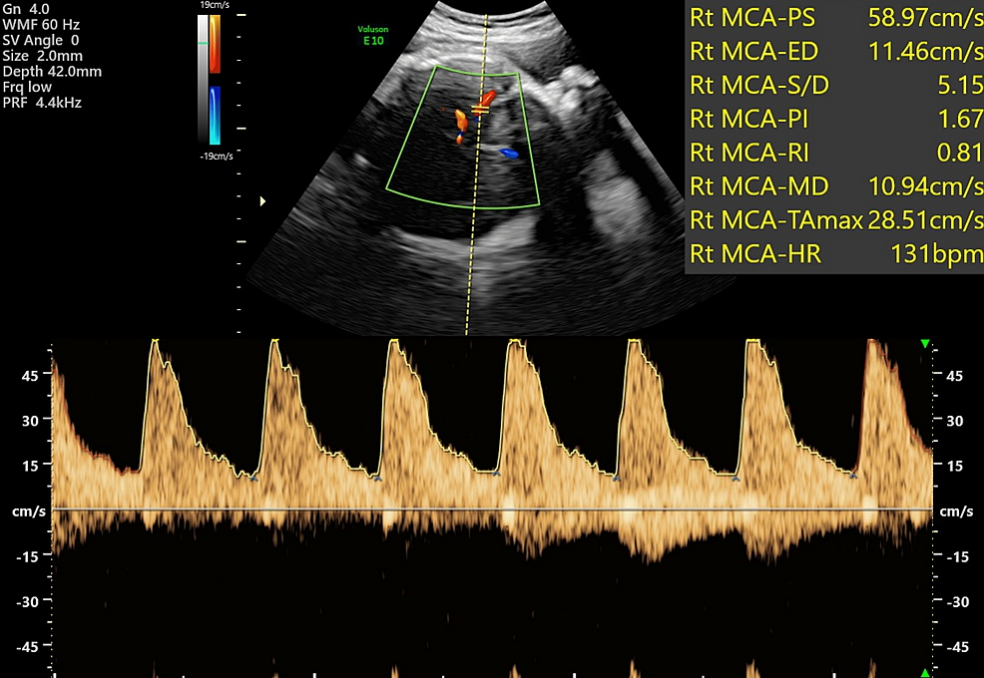
Peak systolic velocity of the middle cerebral artery (MCA) of the foetus at 33 weeks gestation

Ten days later, her FMMU USS scan at 33 weeks and three days showed a live foetus with an EFW of 2457 g and AFI of 14 cm. The chorioangioma had grown and was measuring 14.36 cm x 11.41 cm (Figure 7). The PSV of the MCA was 58.97 cm/sec (Figure 8) indicating mild to moderate anemia.

**Figure 9:**
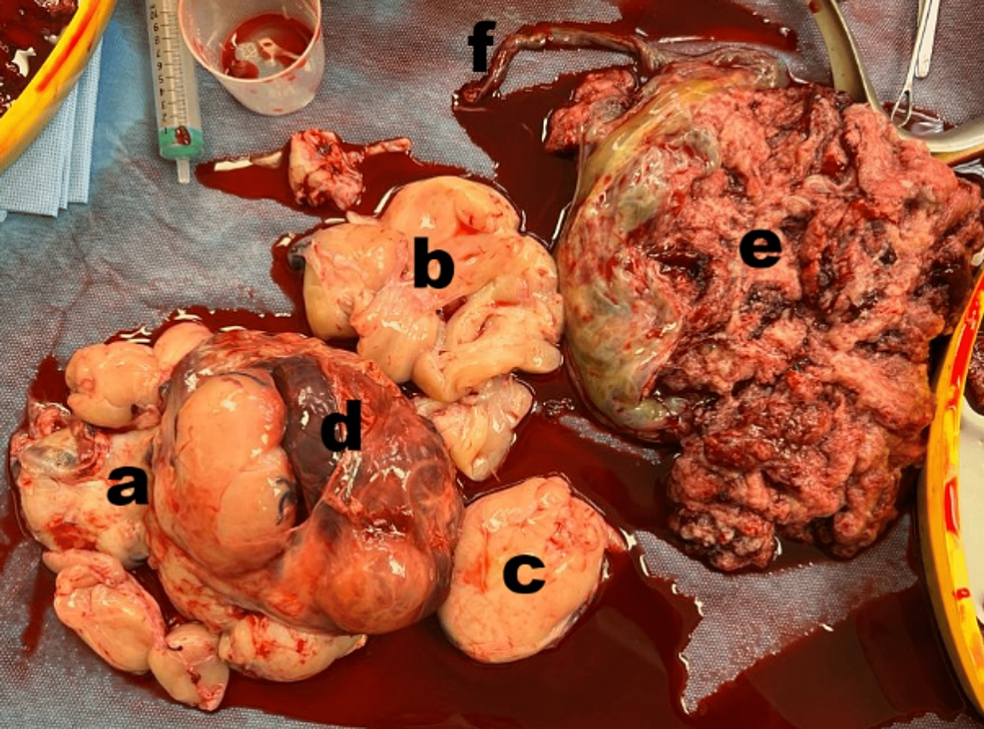
Gross appearance of the chorioangioma and placenta a,b, and c) multiple fragments of tan white lobulated chorioangioma with the largest fragment measuring 14 cm x 10 cm; d) haematoma; e) placenta; f) umbilical cord

At 34 weeks and one day, the patient was admitted due to threatened preterm labor in view of which she was given atosiban and two doses of dexamethasone for fetal lung maturation. The next day she delivered a preterm small for gestational age female baby weighing 1.79 kg via normal vaginal delivery. The neonate’s Apgar score was six and eight at one minute and five minutes, respectively. The liquor was meconium stained so light oral and nasal suction was done. The baby was dusky in color, floppy with irregular breathing, and heart rate >100 bpm but didn't cry after birth, for which she was started on continuous positive airway pressure (CPAP) 5 cm with fraction of inspired oxygen (FiO2) 80% with stimulation for two minutes and after five minutes she was pinkish in color, breathing with good effort and crying with O2 saturation of 93% on 40% CPAP. Then the baby was shifted to the neonatal ICU (NICU) on CPAP 5 cm with 40% FiO2. The mother was taken to the operation theatre for manual removal of the placenta due to retained placenta. After the placenta was taken out and the tumor was removed piecemeal, they weighed 1270 g and 288 g respectively. There was a retroplacental haematoma of 3.5 cm x 3 cm adherent to the subchorionic membrane (Figure 9). Intra-operatively there was approximately 2.5 L of blood loss for which the mother was infused with one litre of sodium chloride, one litre of Ringer's lactate, and one unit of Gelofusin until two units of packed RBC were given. Her cardiovascular support was well managed. Three days later she was discharged with follow-up in two weeks.

After a detailed examination, the neonate was diagnosed with persistent pulmonary hypertension of the newborn, surfactant deficiency causing respiratory distress (was kept on ventilator and CPAP), heart failure with cardiomyopathy and mitral and tricuspid regurgitation confirmed by ECHO, anemia (received three blood transfusions), thrombocytopenia, renal failure and arachnoid cyst in the left frontal lobe as per MRI. The neonate was kept in the NICU for 44 days after which she was discharged.

**Figure 10:**
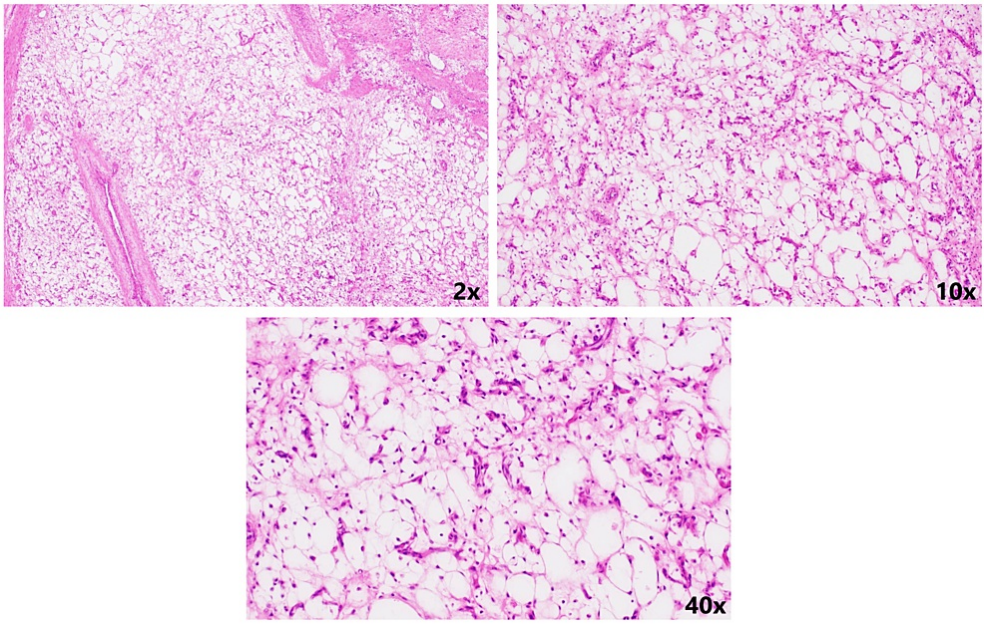
Haematoxylin-eosin stained section shows a loose myxoid stroma at different magnifications

**Figure 11:**
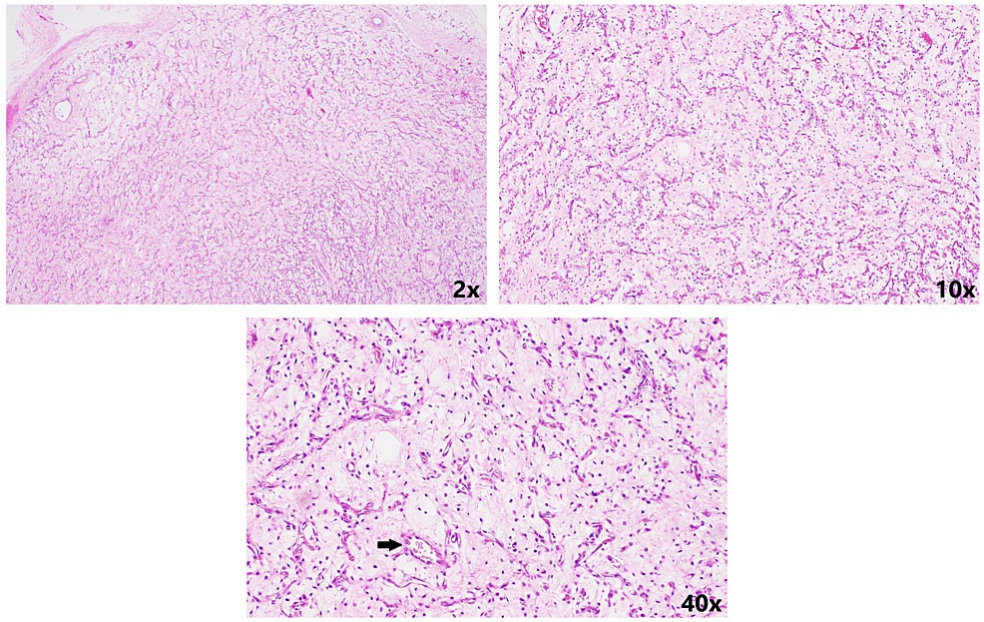
Haematoxylin-eosin stained section shows lobular proliferations of fetal capillaries within the myxoid stroma at different magnifications The endothelial cells of the blood-filled foetal capillaries are arranged in a symmetrical fashion. The red blood cells can be seen in the capillaries. It is clearly visualized at 40x magnification.

Histopathological examination of the placenta showed the placenta corresponding to 34 weeks GA and a chorioangioma of at least 14 cm in size with focal hepatic heterotopia (Figures 10-11); delayed villous maturation with edema at multiple of decidua; moderate meconium-laden macrophages in the amnion; acute chorioamnionitis with stage two neutrophilic infiltration of the chorionic connective tissue; and amnion with presence of grade two chorionic micro-abscesses. A retroplacental haematoma of 3.5 cm x 3 cm was adherent to the sub-chorionic membrane. Immunostaining performed showed retained expression for p57, while the focus of hepatic heterotopia was positive for hepatocyte paraffin 1 (HepPar1), arginase, and alpha fetoprotein (AFP); the chorioangioma endothelial cells were positive for GLUT1 and CD34, and SALL4 was negative. 

Twenty-six days post-delivery the mother came for follow-up with complaints of vaginal bleeding. On transvaginal scan, the uterus was anteverted, anteflexed, and bulky with a large echogenic mass with blood flow which was suggestive of retained placental tissue. USS scan of the pelvis showed a bulky uterus measuring 140 mm x 53 mm x 81 mm with an endometrial thickness of 27 mm containing heterogeneous material with calcific foci and increased peripheral vascularity suspicious of retained placental cotyledon. Then the patient underwent hysteroscopy with dilatation and curettage for the removal of retained placenta tissue. Histopathology confirmed the presence of fragments of viable and necrotic placental tissue, blood clots, and inflamed decidua measuring 6.5 cm x 6 cm x 1.5 cm but no residual chorioangioma. After discharge her follow-up USS was normal with an endometrial thickness of 4.8 mm.

## Discussion

In 1798, J. Clarke diagnosed the first patient with chorioangioma [7,9]. It arises from the mesenchymal tissues of the chorion [5,7]. It is hypothesized that they emerge on day 16 of conception, but there is no record of any case in the first trimester [7-9]. There was a research conducted based on the assessment of 22,000 placentas that disclosed chorioangioma in 138 placentas; it concludes its occurrence as six in 1000 cases [9]. They generally occur in females more than 30 years of age, primigravida, multi-gestation, and with female foetuses [4,7-9]. A retrospective study conducted by Wu and Hu showed that chorioangioma occurred in 70% of foetuses that are female, 80% were primigravidas, and 65% of the tumors caused complications, which is also supported by the research done by Vig et al that shows that the neoplasm affects 75% female foetuses, 60% were primigravida, and it led to maternal and foetal complications in 85% and 50%, respectively [3,10]. Vig et al and Bashiri et al found the mean age of the mother at the time of diagnosis to be 25.4 and 27.5, respectively [10,11].

Chorioangiomas generally grow on the foetal side of the placenta and are coincidentally detected in the third trimester USS with Doppler [4-7]. A grayscale USS detects it as a vascular well-circumscribed mass that projects into the amniotic sac adjacent to the umbilical cord insertion [4-7,9,12]. A systematic review done by Buca et al says that nearly all of the neoplasms are coincidentally detected postnatally and not by the antenatal USS, whereas a few are only diagnosed when they are accompanied by feto-maternal complications [13]. Histopathologically the neoplasm can be classified into three forms, namely, cellular, degenerative, and angiomatous (most common) [1,5,7,9]. Chorioangiomas are grossly encompassed by chorionic epithelium and are well distinguishable from placental tissues [7].

The bigger neoplasms that are more than 5 cm in size behave as arteriovenous (AV) shunts that lead to maternal, foetal, and neonatal complications, such as preterm birth, pre-eclampsia, placenta previa, cervical incompetence, abruptio placentae, post-partum haemorrhage, polyhydramnios, anaemia, hydrops, cardiomegaly, heart failure, thrombocytopenia, disseminated intravascular coagulation, intrauterine growth restriction, intrauterine foetal death, cerebral infarction, and stillbirth [1,4-9,12,14]. Buca et al conducted a systematic review which signified that the occurrence of complications in pregnancy is directly related to the increase in the size of the chorioangioma [13]. The presence of the AV shunt in the neoplasm causes low vascular resistance, which increases the blood flow, leading to a high cardiac output that causes foetal cardiac insufficiency and cardiomegaly, ultimately leading to heart failure [3,6,14-16]. As per McInroy and Kelsey, polyhydramnios develops because the AV shunt in the tumor inhibits the excretion of foetal metabolites into maternal blood, thereby increasing its excretion by the foetal kidney [17]. On the contrary, Wu and Hu believe that the neoplasm presses on the umbilical vein, which leads to increased fetal circulation, which increases urine excretion, leading to polyhydramnios [3]. 

There is intrauterine foetal growth restriction due to the shunting of blood from the villi elsewhere that causes chronic hypoxia, foetal distress, and foetal death [3,15]. Bashiri et al proposed that preterm delivery is seen in 66% of the cases and is caused by the biochemical modification of the amniotic fluid by the tumor [11]. Vig et al and Das et al believe that the collection of platelets and red blood cells in the dead space of the neoplasm lead to microangiopathic haemolysis, which causes foetal thrombocytopenia and anaemia [10,18]. But according to Schmitz et al, chronic haemolysis of the neoplasm led to anaemia, and Kasabach-Merritt type syndrome caused thrombocytopenia. Longstanding anaemia and increased cardiac output led to heart failure soon after birth, whereas the cardio-respiratory collapse was due to the sudden rise in peripheral resistance after the umbilical cord was cut. Cardiac function was further reduced probably due to the blood transfusion that led to volume overload, but that was managed by milrinone [16]. Wu and Hu believe that hydrops is caused as a consequence of thrombocytopenia, anaemia, and cardiac failure, but it is very rare [3]. Das et al reported a case of foetal cerebral infarction that was caused by the prothrombotic factors released from the tumor [18]. In another study done by Kim et al, it was concluded that the infarction of the placental blood vessels and fibrin deposition caused insufficient blood flow to the baby, which led to a cerebral stroke in the baby [19].

Chorioangiomas that are likely to cause complications can undergo treatment before the viability of the developing foetus for a favourable outcome of the pregnancy. The different types of interventions that have been used are thrombosis of blood vessels by alcoholization or endoscopic sutures or endoscopic microcoils or laser coagulation to halt the development of the neoplasm. After foetal development, some therapies that have been used are amnioreduction for polyhydramnios, tocolytic drugs to prevent miscarriage, cordocentesis for foetal anaemia, and steroids for fetal lung maturation before 34 weeks [8,9]. Yet our patient did not undergo any intervention as it is currently not available in our country. A study done by Lim et al states that tumor de-vascularization is recommended when there is foetal hydrops or high output heart failure. Even though there are various interventions, all of them are associated with complications for which USS or foetoscope-guided laser photocoagulation has been used successfully in many of the cases [2].

## Conclusions

Chorioangiomas are benign neoplasms of the placenta. They are generally detected coincidentally on ultrasound during antenatal checkups. Some neoplasms may increase in size as the pregnancy progresses and lead to foeto-maternal complications. Therefore, an early diagnosis warrants a close follow-up and timely intervention for a better outcome of the pregnancy. In our opinion, in order to improve the outcome of the pregnancy in a similar case, the possibility of delivery should be considered based on the size of the chorioangioma, gestational age, and the presence of foetal anaemia.
